# Generation of a novel monoclonal antibody that recognizes the alpha (α)-amidated isoform of a valine residue

**DOI:** 10.1186/s12868-015-0206-y

**Published:** 2015-10-13

**Authors:** Benito Antón Palma, Philippe Leff Gelman, Mayra Medecigo Ríos, Juan Carlos Calva Nieves, Rodolfo Acevedo Ortuño, Maura Epifanía Matus Ortega, Jorge Alberto Hernández Calderón, Ricardo Hernández Miramontes, Anabel Flores Zamora, Alberto Salazar Juárez

**Affiliations:** Molecular Neurobiology and Addictive Neurochemistry Laboratory, National Institute of Psychiatry, Calzada México-Xochimilco #101, 14370 México D.F., Mexico; Department of Neuroscience, National Institute of Perinatology, Montes Urales # 800, 11000 México D.F., Mexico

**Keywords:** Valine, Leucine, Antibody, Antisera, Hybridoma, α-amidation, Cross-reactivity, Immunoassay, Immunoconjugate, Neuropeptide

## Abstract

**Background:**

Alpha (α)-amidation of peptides is a mechanism required for the conversion of prohormones into functional peptide sequences that display biological activities, receptor recognition and signal transduction on target cells. Alpha (α)-amidation occurs in almost all species and amino acids identified in nature. C-terminal valine amide neuropeptides constitute the smallest group of functional peptide compounds identified in neurosecretory structures in vertebrate and invertebrate species.

**Methods:**

The α-amidated isoform of valine residue (Val-CONH_2_) was conjugated to KLH-protein carrier and used to immunize mice. Hyperimmune animals displaying high titers of valine amide antisera were used to generate stable hybridoma-secreting mAbs. Three productive hybridoma (P15A4, P17C11, and P18C5) were tested against peptides antigens containing both the C-terminal α-amidated (–CONH_2_) and free α-carboxylic acid (−COO^−^) isovariant of the valine residue.

**Results:**

P18C5 mAb displayed the highest specificity and selectivity against C-terminal valine amidated peptide antigens in different immunoassays. P18C5 mAb-immunoreactivity exhibited a wide distribution along the neuroaxis of the rat brain, particularly in brain areas that did not cross-match with the neuronal distribution of known valine amide neuropeptides (α-MSH, adrenorphin, secretin, UCN1-2). These brain regions varied in the relative amount of putative novel valine amide peptide immunoreactive material (nmol/μg protein) estimated through a fmol-sensitive solid-phase radioimmunoassay (RIA) raised for P18C5 mAb.

**Conclusions:**

Our results demonstrate the versatility of a single mAb able to differentiate between two structural subdomains of a single amino acid. This mAb offers a wide spectrum of potential applications in research and medicine, whose uses may extend from a biological reagent (used to detect valine amidated peptide substances in fluids and tissues) to a detoxifying reagent (used to neutralize exogenous toxic amide peptide compounds) or as a specific immunoreagent in immunotherapy settings (used to reduce tumor growth and tumorigenesis) among many others.

## Background

Over the past few decades, many neuropeptides have been identified in the neural and neuroendocrine structures of both vertebrate and invertebrate species [1, 2]. In vertebrates, more than 50 % of the identified neuropeptides and peptide hormones are amidated, whereas more than 90 % of bioactive peptides in the brains of invertebrates (i.e., *Drosophila*) are amidated [3]. Most neuropeptides in tissues require post-translational modifications, specifically the α-amidation of the carboxyl group of the C-terminal amino acid. Such amidation represents an essential step for peptide hormones to acquire complete biological activity, receptor recognition, and signal transduction at target cells [4, 5].

**Fig. 1:**
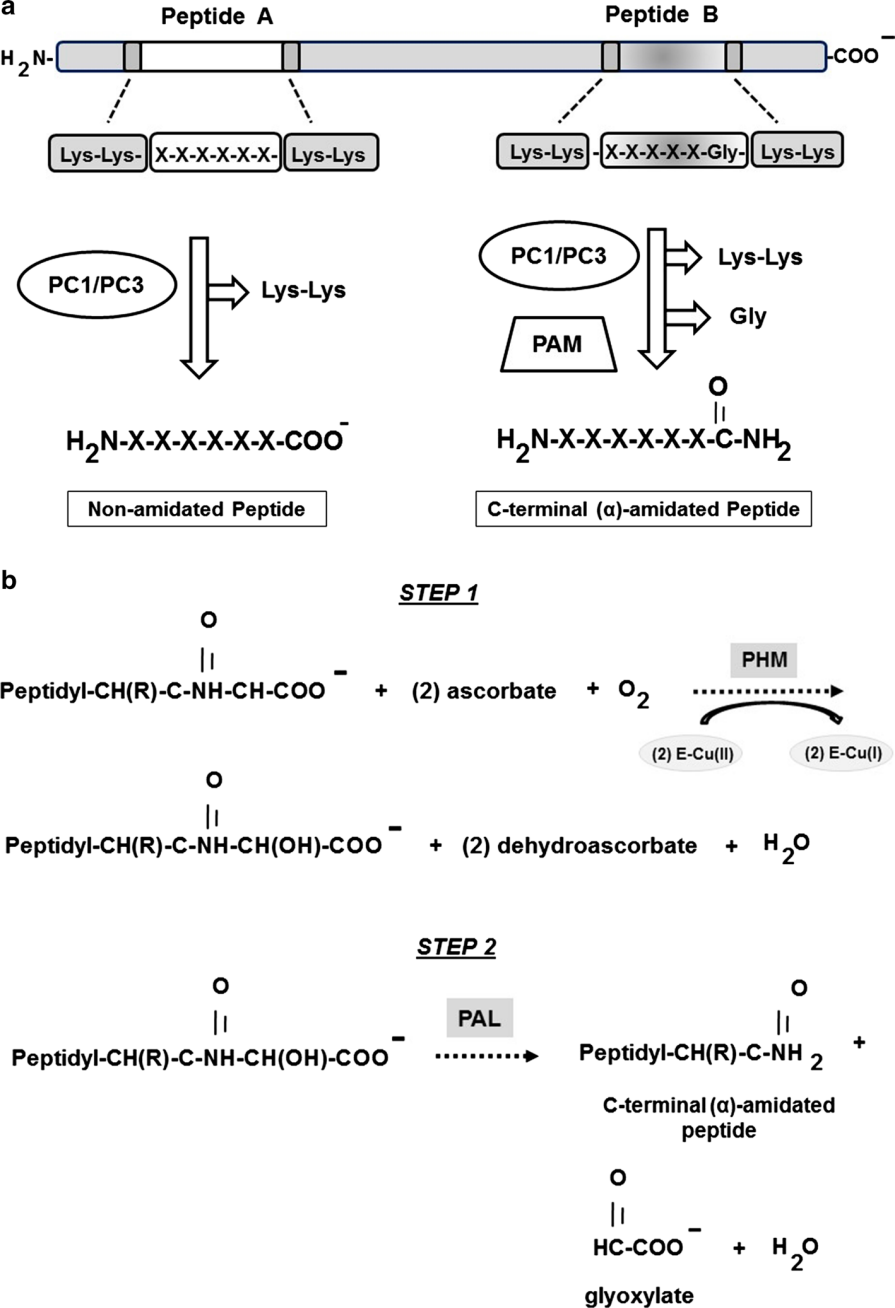
Schematic representation of the α-amidation process in cells. The α-amidation process is a highly specific enzyme-dependent post-translational modification need for converting prohormones into functional peptide products in neurosecretory cells. Panel **a** depicts the specific processing of large propeptide precursor proteins containing active peptide sequences (Peptide A, Peptide B) via PC1/PC3 proconvertase activity alone (Peptide A) or together with the functional activity peptidylglycine α-amidating monooxygenase (PAM) (Peptide B). Panel **b** illustrates the sequential steps of PAM-related bifunctional activity (EC 1.14.17.3) which includes the peptidylglycine α-hydroxylating monooxygenase (PHM) activity depicted in Step 1 and the peptidyl-α-hydroxyglycine-α-amidating lyase (PAL) activity depicted in Step 2. As shown, two molecules of ascorbate are oxidized to form two molecules of semidehydroascorbate during the reduction of two enzyme-bound Cu(II) atoms [E-Cu (II)] into two enzyme-bound Cu(I) atoms [E-Cu (I)] as previously described [10] (for specific details see text in “Background”)

C-terminal amidation of peptides, a necessary step for the conversion of prohormones into functional products, is mediated by the activity of the peptidylglycine α-amidating monooxygenase (PAM), a type I membrane protein that is localized to the trans-Golgi network and to secretory granules in neural and endocrine tissues [6]. PAM is a bifunctional enzyme containing two specific domains: (1) peptidylglycine α-hydroxylating monooxygenase (PHM; EC 1.14.17.3) [7, 8] and (2) peptidyl-α-hydroxyglycine α-amidating lyase (PAL; EC4.3.2.5) [4, 9]. The cellular bioactivity of PAM depends on ascorbic acid, copper, and molecular oxygen [10, 11]. Mice lacking PAM do not survive beyond mid-gestation [12]. Both of its enzymes act sequentially to generate an active α-amidated peptide product and glyoxylate (Fig. 1a–b) [12].

Although a substantial amount of data is available on the reaction mechanism of PHM [7–11] and its relevance to physiology (i.e., memory and learning retention) [13] and medicine (i.e., intermittent hypoxia associated with sleep apnea) [14], few details concerning the activity and relevance of the PAL domain of PAM have been reported [4]. PHM is a di-copper, ascorbate-dependent monooxygenase that catalyzes the stereospecific α-hydroxylation of a glycine (residue)-extended precursor peptide by molecular oxygen (O_2_) [10]; whereas PAL catalyzes the *N*-dealkylation of the peptidyl-α-hydroxyglycine intermediate generated by PHM [10, 11]. In vertebrate species (e.g., human, rat, and *Xenopus*), PHM and PAL are encoded by the same RNA transcript [12, 15], whereas in non-vertebrate species (i.e., *Drosophila*, *Cnidaria*, and *Schistosomes*), each domain appears to be encoded by a separate gene [9].

Although the α-amidation of almost all amino acids found in nature has been observed [1–3, 16], α-amidation of active neuropeptides occurs most frequently on non-polar residues such as phenylalanine (P) (i.e., CCK, FMRF-NH_2_) [16], leucine (L) (i.e., amidorphin) [17], methionine (M) (i.e., bombesin) [2], and glycine (G) (i.e., pancreastatin) [18]. In addition, amidation of some residues such as isoleucine (I) (i.e., CRF, urocortin (UCN)3/stresscopin) occurs rarely [19].

Peptides containing the α-amidated isoform of a valine residue represent the smallest group of functional amide neuropeptides displaying pleiotropic bioactivity (i.e., adaptation and survival, energy and metabolic homeostasis, immunomodulation, and stress and anxiety-related responses) that have been identified in neural structures in both vertebrate and non-vertebrate species [1–3, 16–19]. This group of neuropeptides includes distinct bioactive peptide hormones such as, α-MSH [20], metorphamide/adenorphin [21], secretin [22], urocortins (UCN) [23] and enterins [24].

Early research showed that small and large peptide molecules can be separated via reverse-phase liquid chromatography (RP-LC) based on the hydrophobicity of the peptide structure and its amino acid composition [25]. This separation can be followed by sequence analysis of dansylated amino acids via the Edman technique using dansyl chloride as the reagent to label amino acid residues [26]. These studies led to the discovery of two amidated opioid peptides, adrenorphin and amidorphin [17, 21]. With the advent of recombinant DNA technology and cloning procedures [27] together with proteomic analysis using mass spectrometry techniques (LC/MS/MS) [28], the search for mRNA transcripts and mRNA-translated protein products has led to the identification of many functional peptides [28].

In combination with mass spectrometry, monoclonal antibodies (mAbs) became powerful biological tools used in clinical settings [29] and animal studies [30] to detect post-translational modifications of small molecules in tissues [31]. In the present study, we used splenocyte/myeloma cell fusion technology to generate stable hybridomas secreting mAbs against the structural α-amidated isoform of a valine residue with the goal of identifying and characterizing putative novel C-terminal valine amide neuropeptides in the mammalian CNS and neurosecretory tissues, as well.

Immunohistochemical (IHC) analyses showed that valine amidated neuropeptides, such as, α-MSH [32, 33], metorphamide/adrenorphin [34], secretin [35], and UCN [36] are widely distributed throughout the mammalian CNS and neuroendocrine system [37], including the immune system [38]. In general, antibodies generated to characterize the anatomical distribution of valine-amide peptide immunoreactivity in the brain or neuroendocrine tissues required the synthesis of a fragment or peptide motif encoded along the complete amino acid sequence of the peptide of interest, and used then, as the antigenic epitope to which the antibody is targeted [39]. To date, no IHC studies using specific antibodies raised against small isovariants of a single haptenic molecule, for instance, the free α-carboxylic acid or the structural α-amidated isoform of an amino acid residue (≤200 kDa) have been studied or reported.

Thus, the present paper shows the identification of a single mAb (PC18C5) that displays a high selectivity, sensitivity and specificity for the structural α-amidated isoform of the valine residue when tested against distinct amidated and non-amidated peptide antigens in different immunoassays. Moreover, this mAb exhibited a great capability in detecting endogenous valine amide peptide immunoreactivity along the neuroaxis of the rat brain. Our data demonstrates the high versatility of this mAb, which makes it an amazing biological “tool” for biomedical research and clinical settings.

## Materials and methods

### Peptide synthesis

Synthetic peptides with the primary sequences [Tyr_0_]-*Gly*-*Gly*-*Gly*-Val-CONH_2_ [Tyr- Gly_2-4_-Val-CONH_2_] and [Tyr_0_]-*Gly*-*Gly*-*Gly*-Val-COO^−^ [Tyr-Gly_2-4_-Val-COO^−^] were synthesized by Peninsula Labs (USA). Both isovariants of the valine residue, the α-amidated (Val-CONH_2_) and the free α-carboxylic acid (Val-COO^−^) isoforms, were covalently linked to the C-terminal domain of the peptide backbone and were supplied as lyophilized material at 98 % purity. Both isovariants of the valine residue were used as antigenic epitopes in synthetic peptide constructs and in naturally occurring bioactive peptides to determine the cross-reactivity of the antibodies to each epitope in specific immunoassays. One crucial advantage of using these peptides in immunoassays is that the [Tyr_0_] residue at the N-terminal domain of these synthetic peptides may be used as a radiolabeled tracer in specific radioimmunoassays (RIAs) when the peptides are labeled with [^125^I] (see further details below). Moreover, these short oligopeptide constructs, which were synthesized by condensing a triplet of glycine residues [-Gly(3)-] into a peptide backbone, were designed to maintain optimal backbone flexibility, linear conformation, and solubility in aqueous solution, as has been shown for other polymeric structures containing a short linker backbone of glycine residues [40].

### Immunoconjugates

The structural α-amidated isovariant of the valine residue (Val-CONH_2_; Sigma–Aldrich, USA) was covalently linked to keyhole limpet hemocyanin (KLH, Sigma) according to standard coupling procedures as previously reported [41], and this complex was used as an immunoconjugate for immunization [41]. The free α-carboxylic acid isovariant of valine (Val-COO^−^) (Sigma–Aldrich), the synthetic peptides [Tyr_0_]-Gly_2–4_-Val-CONH_2_ and [Tyr_0_]-Gly_2–4_-Val-COO^−^, the fibronectin/fibrin-related peptide RGDV-COO^−^, metorphamide/adrenorphin (YGGFMRRV-CONH_2_), and the wasp venom-derived ionophoric peptide mastoparan X (INWKGIAAMAKKLL-NH_2_) (Phoenix Pharmaceuticals, USA) were similarly conjugated to bovine serum albumin (BSA) using 0.2 % glutaraldehyde (Sigma–Aldrich, USA) as a cross-linker [41]. The cross-linking reactions were terminated via the addition of 0.1 M glycine (pH 8) to the reaction mixture. The immunoconjugates were dialyzed using a 12 kDa cut-off nitrocellulose membrane (Millipore, USA) for 72 h against 10 L of phosphate-buffered saline (PBS) (pH 7.4) to remove non-reactive aldehydes. Finally, the each immunoconjugate was diluted to 1 mg/mL. The purified immunoconjugates of BSA-linked peptide(s) were frozen and stored at –20 °C for use as adsorbent antigens in ELISAs and dot-blot assays [41].

### Animals

All animal studies and procedures were performed in accordance with the National Institute of Health Guide for the Care and Use of Laboratory Animals (NIH Publication No. 80-23) and were approved by the Animal Care and Bioethics Committee of the National Institute of Psychiatry, Mexico City (NOM -062 -ZOO -1999).

### Immunization

Female BALB/c mice (8–9 weeks old, n = 7) were purchased from Charles River Laboratories (Wilmington, MA) and used for immunization and collection of specific antisera according to standard procedures as described previously [39]. The animals, which were designated as R1–R7, received an initial subcutaneous injection of 0.2 mL of a 1:1 emulsion of 50 μg KLH-Val-CONH_2_ immunoconjugate in 0.1 mL of PBS (pH 7.4):0.1 mL of complete Freund´s adjuvant (Sigma–Aldrich, USA). Three subcutaneous booster injections consisting of 0.2 mL of a 1:1 emulsion of 50 μg KLH-Val-CONH_2_ immunoconjugate in 0.1 mL of PBS (pH 7.4):0.1 mL of incomplete Freund’s adjuvant (Sigma–Aldrich, USA) were administered, followed by a final intraperitoneal booster injection of KLH-Val-CONH_2_ in 0.1 mL of PBS (pH 7.4):0.1 mL of incomplete Freund’s adjuvant 30 days after the previous immunization. Blood (50 μL) was collected from the tail vein of the mice every 2 weeks after each immunization and was centrifuged in an Eppendorf 5804R centrifuge at 1200 rpm for 10 min at 4 °C. The collected sera were stored at −20 °C until further use.

### ELISA for polyclonal antibodies

The titers of polyclonal valine amide antisera (VAA) from vaccinated mice were determined via standard antibody-capture ELISA [41] in microtiter 96-well ELISA plates (Immunolon I, Corning). Each well was coated with 0.3 μg of BSA-[Tyr_0_]-Gly_2–4_-Val-CONH_2_ or BSA-RGDV-COO^−^ immunoconjugate in the presence of 0.1 M sodium bicarbonate. After overnight incubation, the coated wells were blocked with ELISA buffer [0.5 % teleostean gelatin (Sigma–Aldrich, USA), 0.01 M NaH_2_PO_4_, and 0.05 % Tween-20 (Sigma–Aldrich, USA), pH 7.4] for 2 h at room temperature (RT), washed with ELISA buffer, and incubated overnight at 4 °C in 50 μL of VAA diluted 1:10–1:1000. The wells were subsequently incubated for 2 h at RT in 50 μL of PBS (pH 7.4) containing a 1:5000 dilution of horseradish peroxidase (HRP)-conjugated goat anti-mouse immunoglobulin G (IgG) (Jackson Immunoresearch, USA) and 0.05 % Tween-20. Color development was performed using the chromogenic reagent O-phenylenediamine dihydrochloride (OPD, Sigma). The plates were measured at 490 nm in an automated ELISA plate reader (EL 311, Bio-Tek Instruments, USA). Preimmune sera from the mice were used as controls. Each assay was performed in triplicate. Vaccinated mice displaying antisera titers greater than 1:1000 dilution whose sera yielded an A_490_ reading greater than two standard deviations (SD) beyond the mean of the non-specific value obtained using preimmune serum were selected as candidates for cell fusion and for the generation of stable productive hybridomas.

### Cell fusion and generation of productive hybridomas

Hyperimmune mice displaying high antisera titers based on ELISA received an intraperitoneal pre-fusion booster of 50 μg of the KLH-Val-CONH_2_ immunoconjugate in 200 μL of PBS (pH 7.4) in the absence of adjuvant 7 days before lymphoid cells were harvested [41, 42]. Mice were killed by cervical dislocation; splenocytes were collected under aseptic conditions (Biological Safety Cabinet, Nuaire, Class II Type A/B3) and were promptly fused with the murine myeloma Sp2/0 cells (kindly donated by Dr. Pascal Herion, Instituto de Investigaciones Biomedicas, UNAM, Mexico) using 50 % polyethylene glycol 4000 (Gibco)/PBS (pH 7.4) according to standard cell fusion protocols as described previously [41, 42]. The murine myeloma cell line Sp2/0 is a variant line derived from the fusion of MOPC-21 and BALB/c mouse splenocytes [41, 42]; it is frequently used for the generation of stable hybridomas due to its high growth rate, rapid replication rate, high capacity for cell fusion, and cloning efficiency [39, 42]. The fused cells were resuspended at a concentration of 2 × 10^6^ cells/mL in a selective enriched hybridoma medium [RPMI1640 medium (Gibco, USA) supplemented with 20 % fetal calf serum (FCS)(Gibco, USA), 5 % of a complex mixture of growth factors and cytokines BM Condimed H1 Hybridoma Cloning Supplement (Roche, USA), 50 μg of gentamycin (Sigma–Aldrich, USA), 300 μg/mL l-glutamine (Sigma–Aldrich, USA), 15 mM HEPES (Cellgro), and 5 % hypoxanthine, aminopterin, and thymidine (HAT) (Sigma–Aldrich, USA)]. The fused cells were seeded on a thymocyte feeder layer (3.3 × 10^5^ cells/well) in 20 × 96-well plates (Immunolon I, Corning). Myeloma cells and hybridoma colonies displaying logarithmic growth to a density of 5–6 × 10^6^ cells/mL were diluted to 1 × 10^5^ cells/mL using cryopreservation medium (RPMI1640 medium containing 7 % DMSO and 10 % FCS) and stored at −196 °C under liquid nitrogen in cryogenic vials (Nunc).

### ELISA for mAbs

ELISAs were used to identify mAbs against Val-CONH_2_ secreted by positive colonies using the same protocols as those described for the screening of polyclonal antisera from mice except that BSA-Val-CONH_2_, BSA-metorphamide/adrenorphin, BSA-mastoparan-X, BSA-Val-COO^−^, and BSA-RGDV-COO^−^ were used as adsorbent antigens in the wells, that 50 μL of various dilutions (1:50, 1:100, 1:200, 1:400, 1:800, 1:1600, or 1:3200) of the supernatants collected from hybridoma colonies were added to the coated wells, and that the plates were incubated overnight at 4 °C. VAL-CONH_2_-ir signal was detected at 490 nm in an automated ELISA plate reader (EL 311, Bio-Tek Instruments, USA) using a 1:5000 dilution of HRP-conjugated goat anti-mouse IgG (Jackson Immunoresearch, USA) and the chromogenic reagent OPD (Sigma–Aldrich, USA). VAL-CONH_2_ mAb specificity was determined based on the absence of cross-reactivity against the free α-carboxylic acid form of the valine residue (COO^−^) and/or against the C-terminal amide form of the leucine residue (L) in mastoparan X. Preimmune sera from the mice were used as controls in all assays.

### Expansion of hybridomas

Productive hybridomas secreting antibodies were recovered and subjected to expansion and subcloning procedures at limiting cell dilutions as previously described by Hockfield et al. [41].

### Isotyping

Isotyping of the mAbs was performed using an IsoQuick Kit for Mouse Monoclonal Isotyping (Sigma–Aldrich, USA).

### Dot–blot assays

Dot–blot assays were used to assess the mAb specificities against both isovariants of the valine residue in the tested antigens according to standard procedures described by Hockfield et al. [41] and adapted by Loi et al. [43]. Briefly, both isovariants of the valine residue and synthetic BSA-conjugated peptides were tested as potential cross-reactive antigens. Immunoconjugates of the BSA-conjugated antigenic peptides Tyr-Gly_2-4_-Val-CONH_2_, Tyr-Gly_2-4_-Val-COO^−^, RGDV-COO^−^, metorphamide/adrenorphin, and mastoparan X (-Leu-CONH_2_) were diluted in 50 % methanol to obtain stock solutions at concentrations of 10^−2^–10^−9^ M. Two microliters of each antigenic BSA-peptide immunoconjugate at 10^−12^–10^−16^ M were spotted onto nitrocellulose membranes (0.45 μm) (Bio-Rad Labs, USA). After spotting the peptides, the dried membranes were soaked in a preblocking solution containing 1 % teleostean gelatin (Sigma–Aldrich, USA), 10 mM PBS, and 0.05 % Tween-20 (Sigma–Aldrich, USA) (pH 7.4) for 1 h at RT. The membranes were washed 5 times for 5 min with the preblocking solution and incubated overnight at 4 °C in the presence of a 1:25 dilution of hybridoma supernatants containing a mAb in 10 mM PBS (pH 7.4). The membranes were then washed and incubated for 2 h at RT in a solution containing 0.05 % Tween-20 and 10 mM PBS (pH 7.4) in the presence of HRP-conjugated goat anti-mouse IgG (Jackson Immunoresearch, USA) diluted 1:20,000. The membranes were washed 5 times for 5 min with 0.05 % Tween-20 in 10 mM PBS (pH 7.4) to remove excess secondary antibody. Signal detection was performed using a chemiluminescence kit (NEN, Cat. No. NEL-101; Life Sciences, USA).

### Solid-phase RIA

A solid-phase RIA for the α-amidated isovariant of the valine residue was adapted from standard procedures described by Hockfield et al. [41]. Briefly, the N-terminal [Tyr_0_] residue of the synthetic peptide Tyr-Gly_2-4_-Val-CONH_2_ was iodinated with [^125^I] using the IODO-GEN protein labeling procedure (Pierce, USA) according to the manufacturer’s protocol, and the peptide was purified via high-performance liquid chromatography using Hypersil wide-pore 5-μm, C-8, 2 × 150 mm columns. The solid-phase RIA was prepared by loading 100 μL of blocking solution containing 500 ng protein A (Sigma–Aldrich, USA) and 0.1 M NaHCO_3_ (pH 9) in Immunolon II removable wells (VWR), followed by incubation overnight at 4 °C. The coated wells were washed 3 times for 10 min with RIA buffer [0.15 M K_2_HPO_4_, 0.2 % Tween 20 (Sigma–Aldrich, USA), and 0.1 % teleostean gelatin (Sigma–Aldrich, USA), pH 7.4]. Fifty microliters of RIA buffer containing a hybridoma supernatant at 1:20 dilution were added to the wells in quadruplicate, and the plates were incubated overnight at 4 °C (this dilution was previously shown to result in approximately 20–30 % binding). After removing the antibody solution and washing with RIA buffer, 50 μL of RIA buffer containing competitive peptides (0.1 fmol–10 nmol/well in quadruplicate) and the radiolabeled peptide tracer were applied to the adsorbed anti-Val-CONH_2_ mAbs for 2 h at RT. Typical standard displacement curves were generated using approximately 5000 c.p.m. of the radiolabeled tracer [^125^I]-Tyr-Gly_2-4_-Val-CONH_2_ and the non-labeled peptides used in the competitive RIA [Tyr-Gly_2-4_-Val-CONH_2_, BSA-Val-CONH_2_, BSA-Val-COO^−^, RGDV, metorphamide/adrenorphin, and mastoparan X]. The treated wells were washed and counted for 4 min in a 10-channel gamma counter (ISODATA 500, Hewlett–Packard).

### Peptide fractions from neural and endocrine tissues

Tissue extraction and preparation of whole peptide fractions from various neural and endocrine tissues were performed according to standard procedures and the protocols previously described by Asai et al. [44]. Briefly, male Wistar rats (250–300 g, n = 20) pre-anesthetized with lethal doses of sodium pentobarbital (50 mg/kg, i.p.) were decapitated, and the abdominal cavity was opened for surgical extraction of the pancreas and the adrenal glands. Rat brain regions such as the cortex, the hippocampus, the striatum, the hypothalamus, the thalamus, the cerebellum (Cb), the pituitary and the spinal cord and endocrine glands including the adrenal gland and the pancreas were rapidly dissected on ice. The brain regions and the glands were promptly homogenized using a tissue homogenizer (Polytron PT10/35) at 800 rpm in an ice–cold acid:acetone solution (12 N HCl:H_2_0:acetone, 16:6:40, v:v) and centrifuged at 12,000×*g* for 1 h at 4 °C in a Sorvall RC28S centrifuge (DuPont, USA). The HCl:acetone solution was removed from the crude peptide fraction supernatants via vacuum evaporation (Eppendorf Centrifuge Concentrator 5301, Germany). The dried pellets were resuspended in 5 % TFA solution and loaded on Sep-Pak-RP C-18 columns (4 µm, 3.9 × 300 mm) (Waters Inc., USA) pre-activated with 100 % TFA for solid-phase peptide extraction. The peptide fractions from tissue homogenates of the brain and the endocrine glands were eluted from the columns using 20 % TFA:H_2_O, dried via vacuum evaporation and stored at −20 °C. The thawed pellets were resuspended in a solution containing 50 mM Tris–HCl, pH 8.4, and 2.0 mM CaCl_2_, and 50-µL aliquots of RIA buffer containing a 1:5–1:500 dilution of a purified peptide fraction were individually assessed for the P18C5 mAb via solid-phase RIA using the radioactively labeled peptide [^125^I]-Tyr-Gly_2-4_-Val-CONH_2_. The labeled tracer was used to quantify the abundance of Val-CONH_2_-ir in the tissues. Peptide immunoreactivity in the samples is expressed as nmol/μg protein (mean ± standard error of the mean, SEM).

### Protein concentration determination

The protein concentrations in the peptide fractions extracted from the rat brain and neuroendocrine tissues were determined according to standard procedures using the Micro-BCA Protein Assay Kit (Pierce, Rockford; IL, USA; Cat No. 23235) based on the instructions provided by the manufacturer. The absorbance at 570 nm was measured using a microwell plate reader (EL 311, Bio-Tek Instruments, USA). The protein concentration of the samples was expressed as µg/mL (mean ± SEM).

### Tissue preparation

Tissue preparation for IHC detection of Val-CONH_2_-ir was performed according to standard procedures previously described by Anton et al. [45]. Briefly, six male Wistar rats (250–300 g) were anesthetized with sodium pentobarbital (60 mg/kg, i.p.) (Anesket, PISA Lab, Mexico) and transcardially perfused with 250 mL of 10 mM PBS (pH 7.4)/heparin (5 U/mL) solution, followed by perfusion with 800 mL of 4 % paraformaldehyde/10 mM PBS (pH 7.4) solution at 4 °C. The flow rate was held constant (10 mL/min) using a Hamilton double-pumping system (Hamilton, USA). Fixed tissues (from the brain, hypophysis and adrenal glands) were cryoprotected in 30 % sucrose/10 mM PBS (pH 7.4) solution for 3 days at 4 °C. The fixed brain tissues were sliced into 40-μm-thick sagittal sections using a cryostat (Reichter-Jung 3050); alternatively, the fixed neuroendocrine tissues were sliced into 40-μm-thick coronal sections. The sliced sections were collected in 12-well plates (Corning, USA), allowed to float freely in 10 mM PBS (pH 7.4)/0.05 % sodium azide solution, and stored at 4 °C until use. Storage for up to 1 month in the aforementioned solution does not result in loss of immunoreactivity [45].

### Immunohistochemistry

IHC analysis of Val-CONH_2_-ir in brain and neuroendocrine tissue slices was performed according to standard procedures described previously [45] with minor modifications. Briefly, both brain and neuroendocrine tissue sections were processed while free-floating and were initially washed in 10 mM PBS (3 times for 10 min each) followed by 10 % NaBH4 in 10 mM PBS for 15 min. After three additional 10-min washes in 10 mM PBS, the tissue sections were permeabilized with 0.3 % Tween-20 in 10 mM PBS for 20 min and washed with 10 mM PBS (three times for 10 min each). Then, endogenous peroxidase activity was blocked for 40 min in a 0.3 % hydrogen peroxidase/0.1 % Tween-20/10 mM PBS (pH 7.4) solution. Following three additional 10-min washes in 10 mM PBS, the sections were incubated for 4 h in a preblocking solution containing dialyzed 10 % horse serum, 1 % BSA, 0.3 % Tween-20, and 10 mM PBS (pH 7.4). The sections were then incubated on an orbital shaker for 16 h at 4 °C in the primary valine amide MAb P18C5 diluted 1:40 in the preblocking solution [10 % dialyzed horse serum, 0.3 % Tween-20, and 10 mM PBS (pH 7.4)]. After 5 10-min washes with 10 mM PBS, the sections were incubated for 2 h in a donkey anti-mouse biotinylated (IgG) secondary antibody (Jackson Immunoresearch, USA) diluted 1:2000 in pre-blocking solution [5 % dialyzed horse serum, 1 % BSA, 0.3 % Tween-20, and 10 mM PBS (pH 7.4)]. After 1 and 5 10-min washes with the preblocking solution and with 0.3 % Tween-20/10 mM PBS (pH 7.4) solution, respectively, the sections were incubated in the avidin–biotin complex (ABC Elite Vector Kit; Vector, Burlingame, CA), followed by 310-min washes with 0.1 % Tween-20 in 10 mM PBS and by 3 10-min washes with 10 mM PBS (pH 7.4). Color development was enhanced by a 5–10 min incubation in a 0.06 % 3,3′-diaminobenzidine tetrahydrochloride (DAB, SIGMA)/0.03 % H_2_O_2_/0.3 % nickel sulfate/10 mM PBS (pH 7.4) solution. The sections were then washed in 10 mM PBS, mounted on slides, and air-dried overnight. The prepared slices were cleaned with xylene and cover-slipped with Entellan (Merck). Microscopic analysis of the brain sections was performed under bright-field illumination using a DAS LEICA DMR Qwin microscope. Positive immunoreactivity detected by the PC18C5 mAb in tissue slices is referred as valine amide-like immunoreactivity (VAL-CONH2-ir). The neuroanatomical areas displaying positive VAL-CONH_2_-ir signal were identified according to the atlas of the rat brain by Paxinos and Watson (1998) [46].

### Controls

**Fig. 2:**
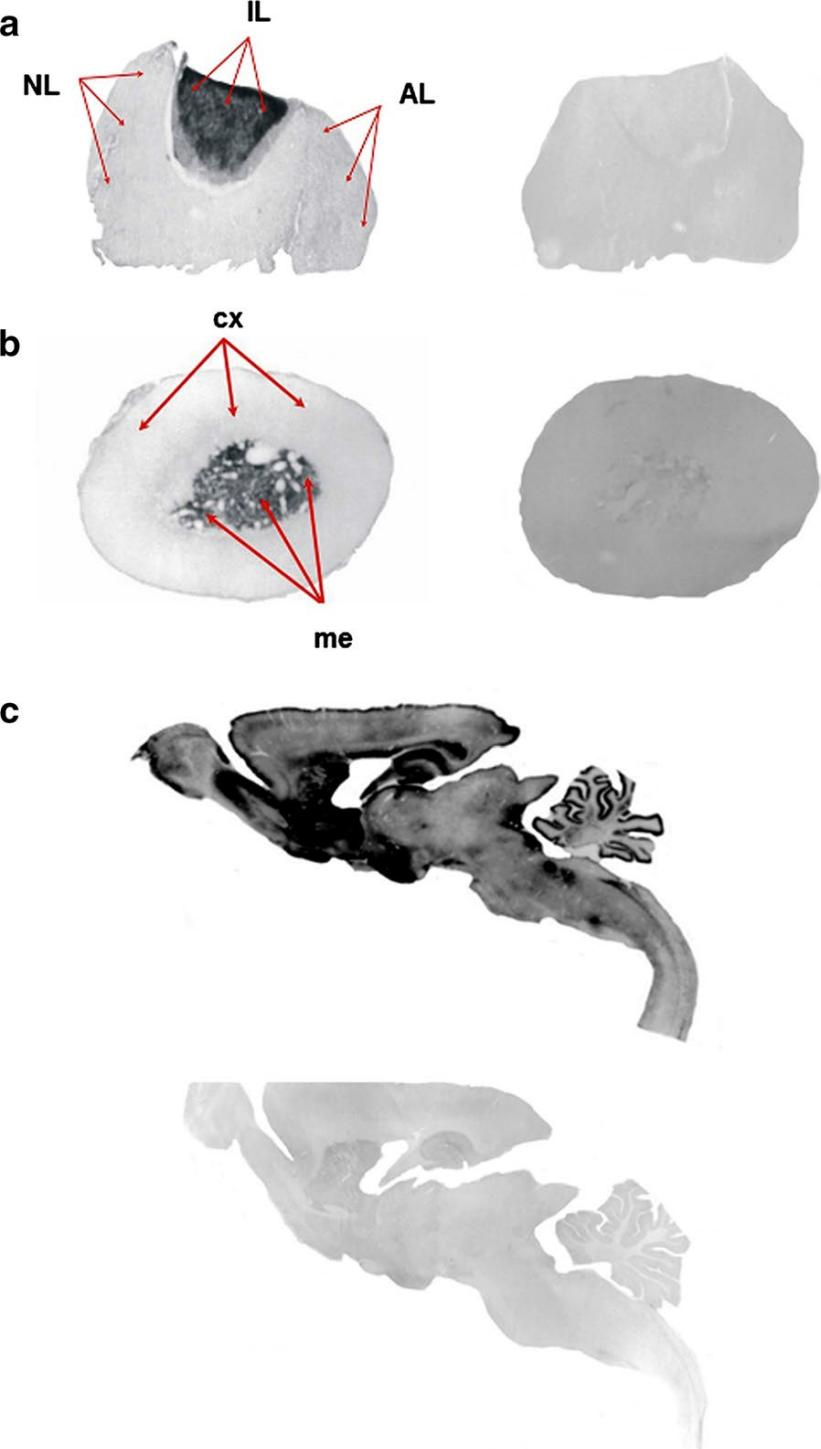
Pre-absorption control assays of valine amide immunoreactivity in tissues. The panels show bright-field photomicrographs (×40) of Val-CONH_2_–ir in neuroendocrine glands and sagittal sections of the rat brain using the specific PC18C5 mAb. Panel **a** illustrates the expression of Val-CONH_2_–ir in the pituitary gland before (*left panel*) and after pre-absorption with 10 μM of the isovariant of the valine residue (Val-CONH_2_) (*right panel*). Note the absence of PC18C5 mAb-ir in the tissue after treatment with the Val-CONH_2_ residue. *NL* neural lobe, *IL* intermediate lobe, *AL* anterior lobe (*red arrows*). Panel **b** illustrates the expression of Val-CONH2-ir in the adrenal gland before (*left panel*) and after pre-absorption with 10 μM of the isovariant Val-CONH_2_ (*right panel*). PC18C5 mAb-ir was localized to the core region of the adrenal gland (me) (*red arrow*). Panel **c** shows the expression of Val-CONH2-ir in a representative sagittal section of the rat brain before (*left panel*) and after pre-absorption with 10 μM of the isovariant Val-CONH_2_ (*right panel*). The intensity of immunoreactive signals was arbitrarily graded using a scale previously described in [45] (see text for additional details). *Scale bar* = ×40

As a control of labeling specificity, adjacent slices from rat neuroendocrine tissues and brain sections were preabsorbed with 10 μM of the valine amide residue (Val-CONH_2_) (Peninsula Labs, USA). The intensity and the density of positive PC18C5 mAb-immunoreactive signal in the brain were compared to those of Val-CONH_2_-ir detected in the pituitary and adrenal glands as shown in Fig. 2a–c. Both tissues were as used internal standards, and the immunoreactive signals were arbitrarily graded using a similar grading scale to the scale used to map the distribution of ORL-1 receptor immunoreactivity in the rat brain [45] as follows: very intense (++++); intense (+++); moderate (++); low (+); and not detected (0).

### Subtraction analysis

**Fig. 3:**
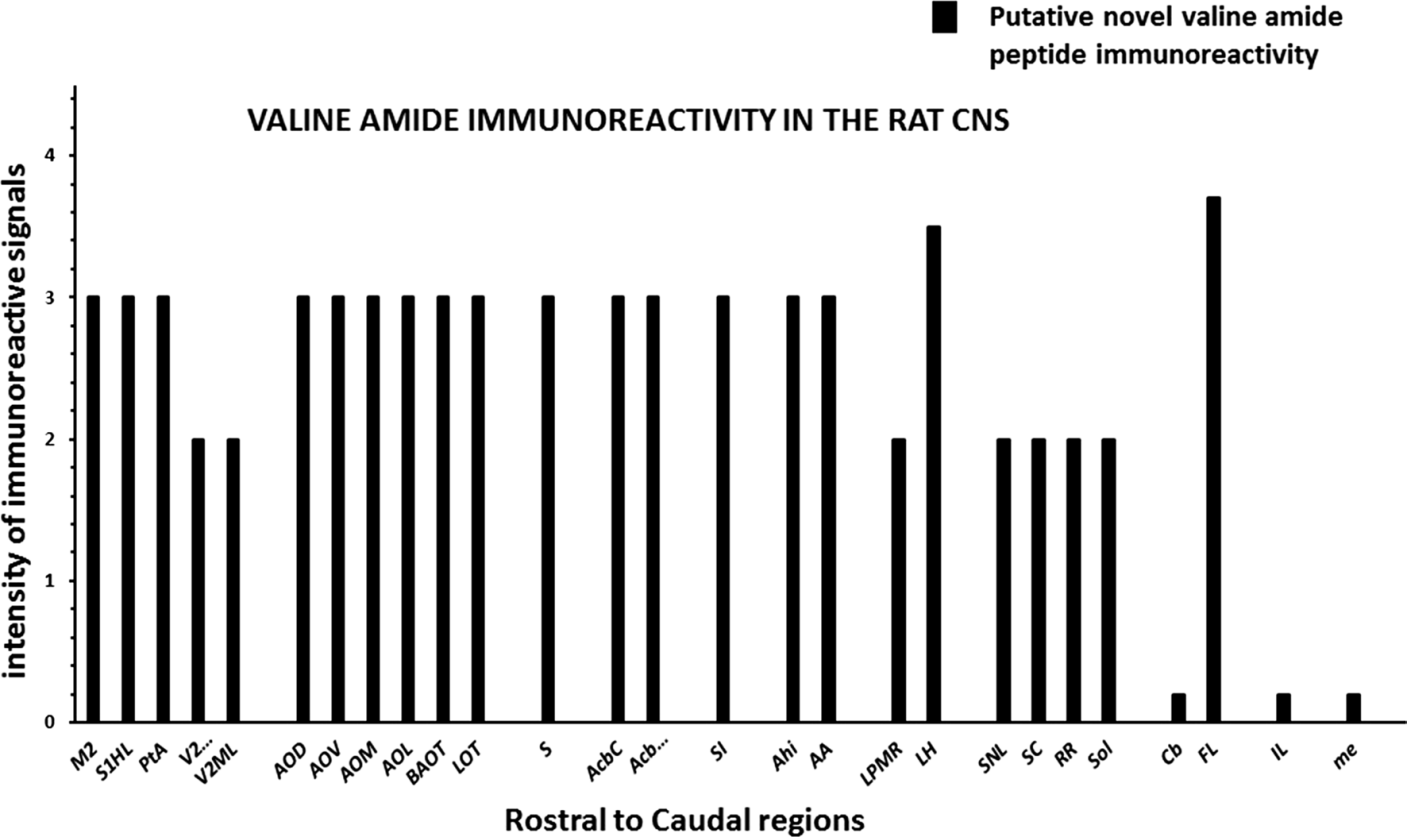
Subtracted brain regions expressing putative valine amide peptide immunoreactivity. P18C5-mAb-ir detected along the neuroaxis of the rat brain (Fig. 7a–e) was subtracted from brain areas expressing peptide immunoreactivity for α-MSH, metorphamide, secretin, and urocortins 1-2. As shown, moderate-to-high intensity mAb-ir signals detected in subtracted brain areas were localized to the cortex [secondary motor (M2), the hindlimb (S1HL), the trunk region (S1Tr) of the primary somatosensory, parietal association (PtA), secondary visual mediomedial (V2MM) and mediolateral (V2ML) cortices] and the main and accessory olfactory nuclei [anterior accessory olfactory nucleus dorsal (AOD), nucleus ventral (AOV), nucleus lateral (AOL) and nucleus medialis (AOM), the olfactory tubercle (Tu), the bed nucleus of the accessory olfactory tract (BAOT) and the nucleus of the lateral olfactory tract (LOT)]. Subcortical areas displaying mAb-ir signal included the subiculum (S), the core (AcbC) and shell (AcbSh) of the nucleus accumbens (NACC), the substantia innominata of the septum (SI), the amygdalo-hippocampal (Ahi) and amygdalo-anterior nuclei (AA) of the limbic system, the lateral posterior nucleus of the thalamus (LPMR) and the lateral hypothalamic (LH) nuclei. Brainstem areas included the substantia nigra (SNL), the superior colliculus (SC), the retrorubral nucleus (RR), the solitary tract (Sol) and the flocculus of the cerebellum (FL). With the exception of the areas described above, only the LH and the FL showed intense immunolabeling similar to that detected in cortical layers of the Cb, the IL of the pituitary and the adrenal medulla (me). As shown, these brain areas were arbitrarily graded as displaying low Val-CONH_2_-peptide immunoreactive signals because they corresponded to areas containing the amidated peptides used as references in this study (see text for additional details). The neuroanatomical areas showing putative valine amide peptide immunoreactivity were identified based on the rat brain atlas of Paxinos and Watson [46]

Immunoreactive signals produced from peptide antibodies used to map the anatomical distribution of C-terminal valine amide neuropeptides [i.e., metorphamide, secretin, α-MSH, and UCNs] in the rat CNS and neuroendocrine tissues were used as a reference to subtract from PC18C5 mAb-ir signals detected in the rat CNS. Positive PC18C5 mAb-ir signals in brain areas that did not match with previously identified C-terminal amide–peptide immunoreactivity, were considered as potential brain areas expressing putative novel valine amide neuropeptides. Positive Val-CONH_2_-ir signals detected in subtracted brain regions were arbitrarily graded as follows: very intense (4); intense (3); moderate (2); low (1); or not detected (0) (see Fig. 3).

### Statistical analysis

Student’s *t* test was used to establish significant differences between samples assessed via RIA or ELISA. The significance level was set at P ≤ 0.05.

## Results

### Identification of hyperimmune vaccinated mice

**Fig. 4:**
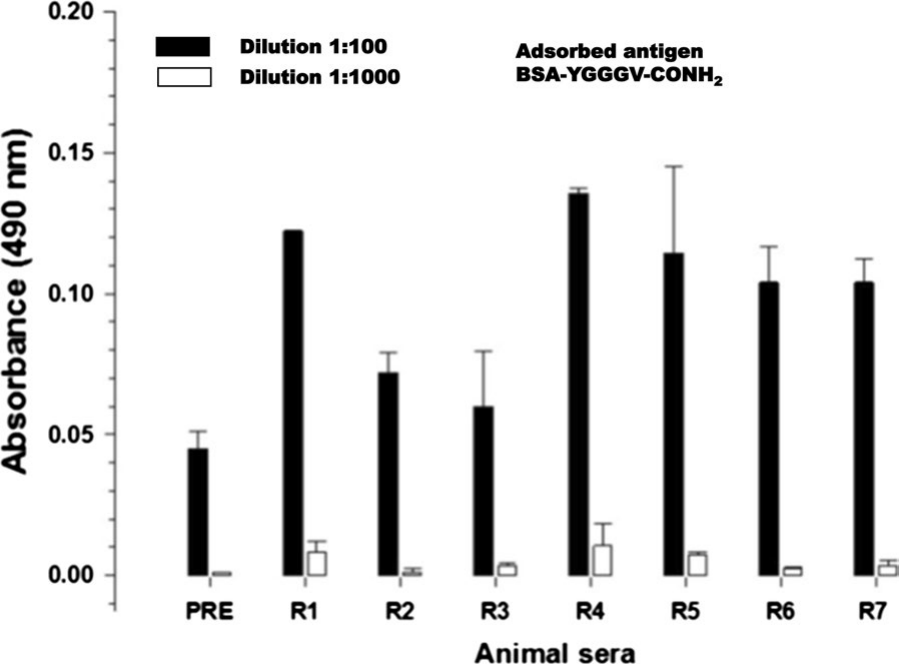
Identification of valine amide antibodies in mice. Animals defined as R1–R7 (female BALB/c mice, 8–9 weeks, n = 7) were vaccinated with an initial subcutaneous injection with a 1:1 emulsion of 50 µg of keyhole limpet hemocyanin (KLH)-Val-CONH_2_ immunoconjugate and complete Freund’s adjuvant, followed by booster injections with incomplete Freund’s adjuvant. Sera were collected and used to identify high-titer valine amide antisera (VAA) in the vaccinated animals using a standard ELISA. The wells were coated with synthetic immunoconjugates of bovine serum albumin (BSA)-[Tyr_0_]-Gly_2–4_-Val-CONH_2_ or BSA-RGDV-COO. The BSA-RGDV-COO^−^-coated wells displayed no significant signal (data not shown, see text for additional details). Assays were performed in triplicate using two serial dilutions of mouse antisera (1:100; 1:1000). The ordinate describes the immunoreactive signals detected as absorbance at 490 nm (A_490_) in the assay. The values are expressed as the mean ± SEM

Immunization of female BALB/c mice (8–9 weeks, n = 7) with the KLH-Val-CONH_2_ immunoconjugate after five consecutive booster injections produced animals exhibiting antibody responses against the α-amidated isoform of the valine residue (Val-CONH_2_) (Fig. 4). VAAs collected from five (R1, R2, R4, R6, and R7) of the seven vaccinated mice displayed significantly high cross-reactive signal against the BSA-Tyr-Gly_2-4_-Val-CONH_2_ adsorbed antigen based on ELISA; these signals were twofold higher than the non-specific A_490_ values detected for the pre-immune control serum at the tested dilutions (1:100–1:1000). However, the VAAs from the vaccinated animals did not display significantly higher cross-reactive signal than the controls for the free α-carboxylic acid isoform of the valine residue in the BSA-RGDV-COO^−^ antigen (data not shown). The criteria for the selection of the samples for the cell fusion procedures were based on the detection of high immunoreactive signals (A_490_ values) against the α-amidated isoform of the valine residue in the BSA-Tyr-Gly_2-4_-Val-CONH_2_ immunoconjugate at the highest antisera dilution tested (1:1000) in vaccinated animals and on the lack of immunoreactivity for the carboxylic acid form of the same residue in the BSA-RGDV-COO^−^ antigen (see the “Materials and methods” section). Among the five hyperimmune animals (R1, R2, R4, R5, and R7) selected for the generation of hybridoma-secreting mAbs, mouse R4 exhibited the highest cross-reactivity against BSA-Tyr-Gly_2-4_-Val-CONH_2_, displaying high titers of antibodies at VAA dilutions of 1:100 (A_490_ = 0.062 ± 0.002, mean ± SEM) and 1:1000 (A_490_ = 0.0034 ± 0.0002; mean ± SEM) (Fig. 4).

### Productive hybridomas

Cell fusion of activated splenocytes from mouse R4 with the murine myeloma Sp2/0 cells yielded 340 stable hybridomas (17.8 %) from a total of 1920 seeded cells (data not shown). ELISA revealed three stable hybridoma-forming colonies (0.0015 %), designated as P15A4, P17C11, and P18C5; these three colonies secreted mAbs that recognized the cognate epitope of Val-CONH_2_ in both the BSA-Val-CONH_2_ and BSA-Tyr-Gly2-4-Val-CONH_2_ immunoconjugates (data not shown). The hybridomas secreting mAbs displaying cross-reactivity against the α-amidated isoform of the valine residue represented 8.8 % of the total productive hybridomas formed after cell fusion. Isotyping of the immunoglobulin class of the mAbs secreted by these three hybridomas showed that they belonged to the IgG1 class.

### ELISA

ELISAs were evaluated as an initial step in characterizing the specificity of the three distinct antibodies (P15A4, P17C11, and P18C5) against the structural α-amidated form of the valine residue. Immunoconjugates of amidated and non-amidated Val- and Leu-containing peptides linked to BSA were used as adsorbed antigens in the assays (see the Materials and methods section). The P15A4 and P17C11 mAbs displayed no differences in cross-reactivity against the adsorbed antigens of BSA-Val-CONH_2_, BSA-Tyr-Gly_2-4_-Val-CONH_2_, metorphamide (YGGFMRRV-CONH_2_), BSA-Val-COO^−^, or mastoparan X (INWKGIAAMAKKLL-CONH_2_) at the tested antibody dilutions (1:50–1:6400) (data not shown).

**Fig. 5:**
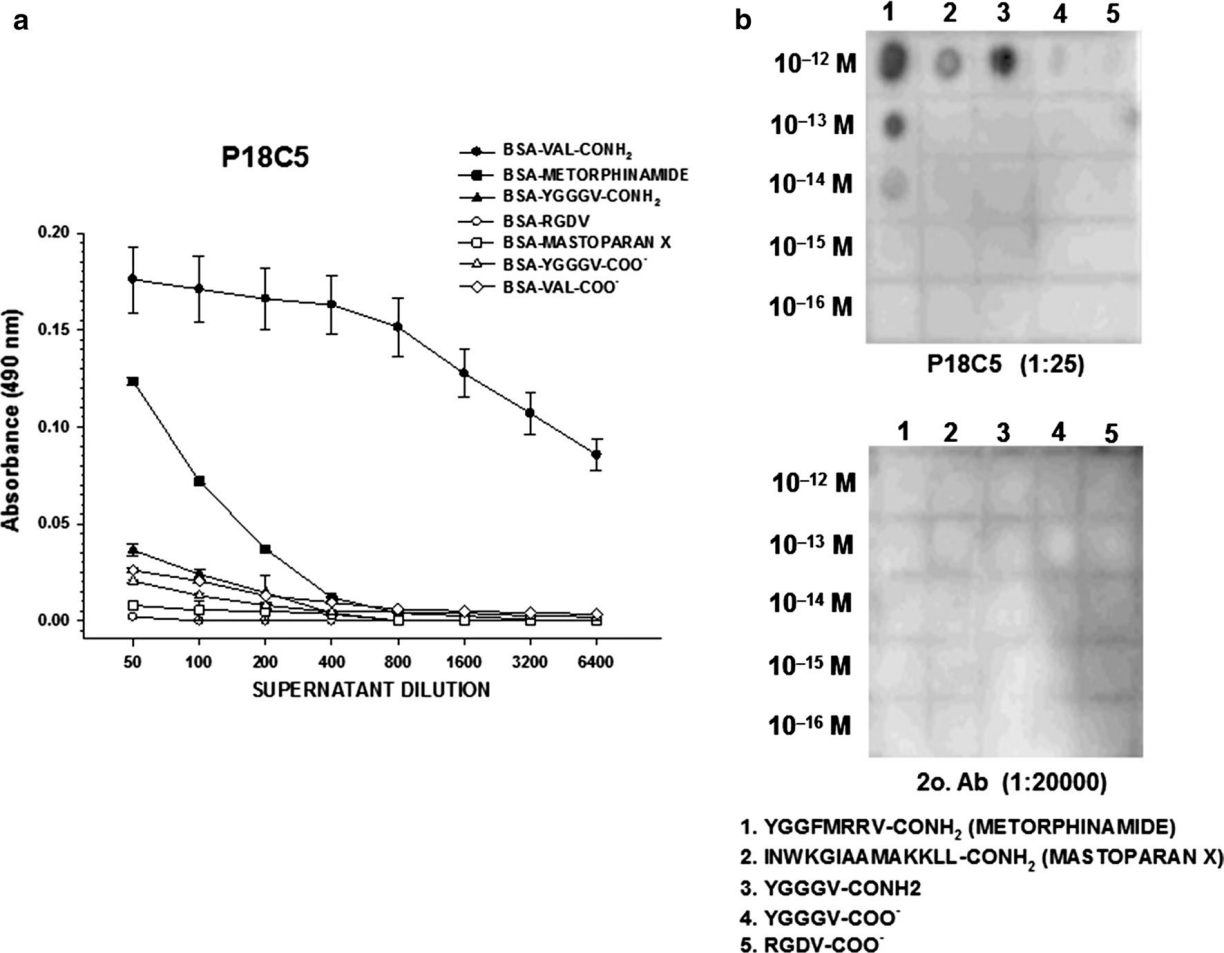
Characterization of P18C5 mAb specificity. The vaccinated animals displaying the highest titers based on ELISA were used for the generation of stable productive hybridomas. Supernatants from the resulting hybridoma-producing colonies were screened via standard ELISA to assess the cross-reactivity of hybridoma-secreting mAbs against the isovariants of the Val (V) or Leu (L) residues contained in the C-terminal domain of BSA-conjugated peptides and were used as adsorbed antigens together with BSA-Val-CONH_2_ and BSA-Val-COO^−^ immunoconjugates in the assay. The P18C5 mAb was tested at increasing dilutions (1:50–1:6400) in ELISAs (**a**) or at a specific dilution (1:25) in dot-blot assays (**b**, *upper panel*). BSA-Val-CONH_2_, BSA-Val-COO^−^ and BSA-(Val/Leu) peptide immunoconjugates were used as adsorbed antigens in the ELISA or as spotted antigens (10^−12^–10^−16^ M) in the dot–blot assay. ELISA (**a**) was performed in triplicate using serial twofold dilutions of the P18C5 mAb (abscissa). The absorbance of the immunoreactive signals in positive wells (ordinate) was measured at λ = 490 nm (see text for additional details)

However, the P18C5 mAb displayed a distinct pattern of reactivity against the α-amidated and free carboxylic acid isovariants of the valine residue in the tested antigens, as depicted in Fig. 5. As shown, the P18C5 mAb exhibited high evident cross-reactivity against the α-amidated form of the valine residue in BSA-Val-CONH_2_, at both low and high antibody dilutions (1:50–1:6400). Notably, a less apparent immunoreactive response was detected against the metorphamide/adrenorphin peptide antigen. Cross-reactivity with the latter antigen decayed rapidly at the initial working dilutions of the antibody (1:50–1:200), in contrast to the slow decay of immunoreactive signal for the BSA-Val-CONH_2_ antigen at higher dilutions (1:800). However, the P18C5 mAb cross-reacted with the antigenic isovariants of the valine residue in the BSA-Val-COO^−^, BSA-Tyr-Gly_2-4_-Val-CONH_2_, BSA-Tyr-Gly_2-4_-Val-COO^−^, and BSA-RGDV-COO^−^ immunoconjugates, including the α-amidated isoform of Leu in the mastoparan X peptide antigen. The immunoreactive signals against these antigens were 4.5- and 21.4-fold lower than those against the BSA-Val-CONH_2_ antigen at the lowest (1:50) and highest (1:6400) antibody dilutions, respectively.

### Dot–blot assays

Dot–blot assays were used to confirm and validate the specificity of the selected antibodies (P15A4, P17C11, and P18C5) against the conjugated BSA-peptide antigens (Metorphamide, Mastoparan X, Tyr-Gly_2-4_-Val-CONH_2_, and Tyr-Gly_2-4_-Val-COO^−^) tested in the assay (Fig. 5b). In general, the results of the dot–blot assays showed that the intensity of IHC staining from each hybridoma-secreted mAb was proportional to the concentration of the peptide antigen spotted on the membrane filter (1 × 10^−12^–1 × 10^−16^ M). The intensity of the detected immunoreactive signals in the dot–blots was compared to that of the control filters using only a secondary mouse-IgG antibody, as depicted in Fig. 5b.

As shown, P18C5 mAb (as opposed to the nonspecific reactive signals generated by the P15A4 and P17C11 mAbs) displayed a high specificity and selectivity for the α-amidated isoform of the valine residue among the peptide antigens tested in the assay, as shown in Fig. 5b. This mAb exhibited very intense immunoreactive signals against the metorphamide and YGGG-Val-CONH_2_ peptide antigens at the highest peptide concentration tested (10^−12^ M), showing moderate immunoreactive signals against the peptide at lower peptide concentrations (10^−13^–10^−14^ M). However, a weak immunoreactive signal was observed against Mastoparan X at the highest concentration tested (10^−12^ M), and no signals were observed at lower concentrations (10^−13^–10^−14^ M). Interestingly, no immunoreactive signals against both YGGG-Val-COO^−^ and RGDV-COO^−^ antigens were detected at any of the peptide concentration tested. Thus, the P18C5 mAb showed superior antibody selectivity against the α-amidated form of the valine residue.

Based on the high specificity and selectivity of the P18C5 mAb for the α-amidated form of the valine residue in peptide antigens based on ELISAs and dot–blot assays, a sensitive solid-phase RIA for P18C5 mAb was developed and used to further assess the specificity and selectivity of this hybridoma-secreted mAb. Validation of the sensitivity of this RIA enabled further identification and characterization of putative novel valine–amide neuropeptides from brain and neurosecretory tissues of rodents in subsequent studies.

### Solid-phase RIA

**Fig. 6:**
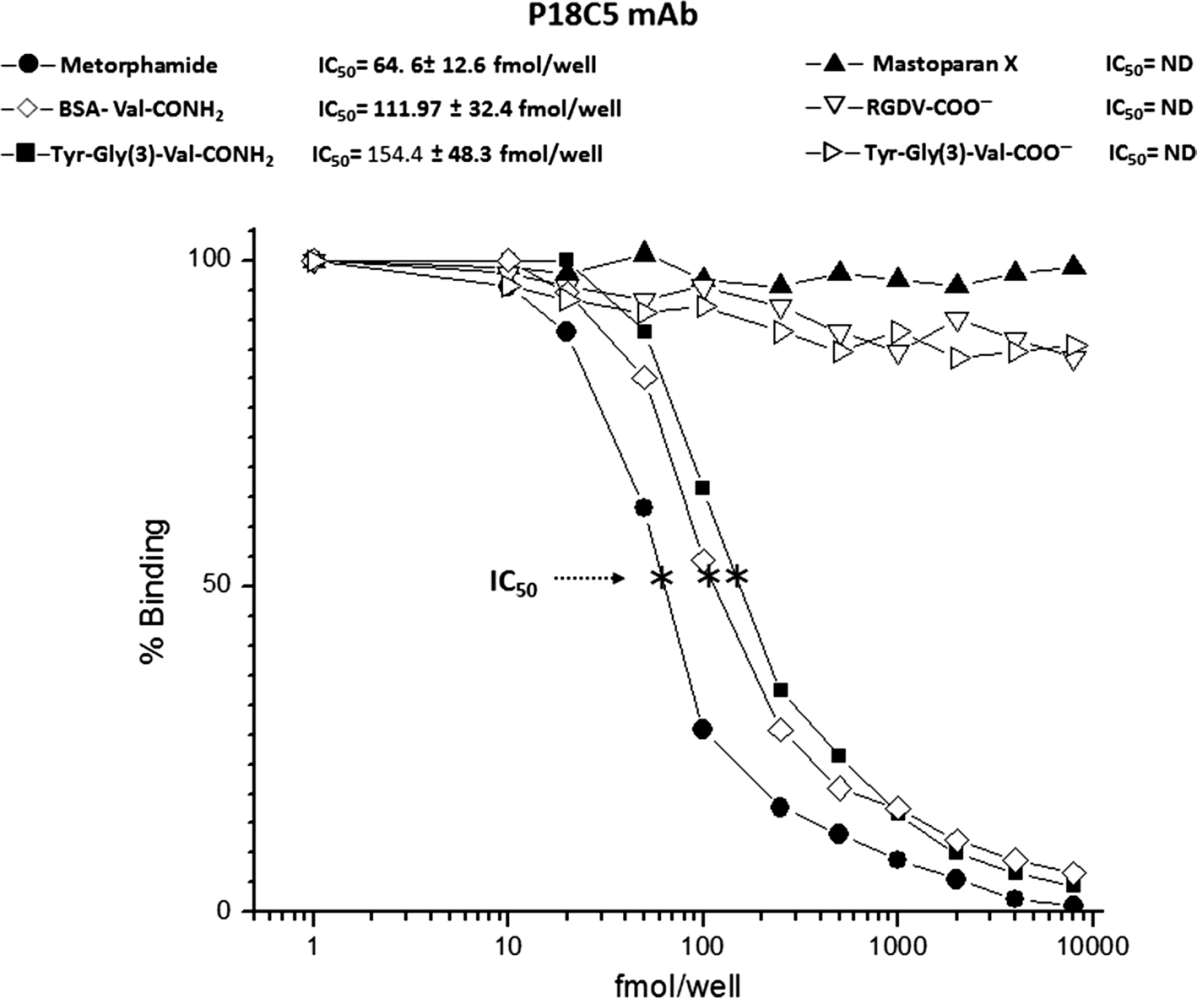
Specific solid-phase radioimmunoassay for the P18C5 mAb. The P18C5 mAb was used to generate a reliable and sensitive solid-phase RIA for detecting the structural α-amidated isoform of the valine residue in synthetic peptide and non-peptide antigens. As shown, the P18C5 mAb was highly sensitive in detecting the VAL-CONH_2_ residue in metorphamide/adrenorphin, BSA-VAL-CONH_2_ and Tyr-Gly_2-4_-Val-CONH_2_ antigens within the tested concentration range, displaying IC_50_ values of 64.6 ± 9.7 fmol/well, 111.97 ± 32.4 fmol/well, and 154.4 ± 48.3 fmol/well, respectively. However, no cross-reactivity (IC_50_ = ND) was detected against antigens expressing the Val-COO^−^ residue in Tyr-Gly_2-4_-Val-COO^−^ or RGDV-COO^−^, including mastoparan X, within the range of competitive antigen concentrations (0.1 fmol–10 nmol) tested in the assay (see text for additional details)

A fmol-sensitive solid-phase RIA for the P18C5 mAb was developed using both the structural α-amidated isoform and the free carboxylic acid form of the valine residue conjugated to BSA, including both the C-terminal amide peptides and synthetic non-amidated peptides, using the synthetic [^125^I]-Tyr-Gly_2-4_-Val-CONH_2_ as the labeled tracer (see the Materials and methods section). A representative RIA showing typical displacement curves by synthetic [^125^I]-Tyr-Gly_2-4_-Val-CONH_2_ using the P18C5 mAb is depicted in Fig. 6. As shown, the P18C5 mAb recognized non-labeled Tyr-Gly_2-4_-Val-CONH_2_ over the tested concentration range, with an IC_50_ of 154.4 ± 48.3 fmol/well; the smallest measured displacement value, detected at IC_80_, was 64.8 ± 11.6 fmol/well, and the highest was 710.2 ± 89.8 fmol/well at IC_20_ based on this assay. In the same context, metorphamide was bound at an IC_50_ of 64.6 ± 9.7 fmol/well, and its lowest and highest displacement values were detected at the IC_80_ (26.9 ± 7.3 fmol/well) and the IC_20_ (179.7 ± 44.8 fmol/well), respectively. Similar displacement values were observed for BSA-VAL-CONH_2_. The P18C5 mAb detected BSA-VAL-CONH_2_ at an IC_50_ of 111.97 ± 32.4 fmol/well, an IC_80_ of 53.7 ± 12.7 fmol/well, and an IC_20_ of 458.56 ± 69.9 fmol/well. Moreover, no significant cross-reactivity against mastoparan X was observed for the concentration range of competitive peptides (0.1 fmol–10 nmol) tested in this assay. Furthermore, the P18C5 mAb failed to recognize the structural free carboxylic acid isoform of the valine residue in both Tyr-Gly_2-4_-Val-COO^−^ and RGDV-COO^−^ over the tested concentration range of the competitive peptide antigens (IC_50_, non- determined), except for the lowest measurable displacement values detected at the IC_80_ for Val-COO^−^ peptides (>2152.1 ± 469.6 fmol/well) and for mastoparan X (>8125.2 ± 1675.8 fmol/well).

### P18C5 mAb immunoreactivity in control tissues

Isotyping analysis of the P18C5 mAb showed that it is classified as an IgG1 antibody. As shown in Fig. 2a–c, P18C5 mAb immunostaining was abolished in control tissues of the hypophysis, the adrenal gland, and representative sagittal sections of the rat brain when the antibody was pre-absorbed with 10 μM of the synthetic Val-CONH_2_ compound. The intermediate lobe of the hypophysis (IL) displayed intense P18C5 mAb immunolabeling (Fig. 2a, left panel). Pre-absorption with the synthetic amidated valine compound completely abolished P18C5 mAb staining in this region (Fig. 2a, right panel). Furthermore, the chromaffin cell-rich area of the adrenal gland [the adrenal medulla (me)] displayed intense P18C5 mAb immunolabeling (Fig. 2b, left panel). Pre-absorption with the synthetic amidated valine compound completely blocked immunostaining in the core area of the adrenal gland (*me*) in adjacent tissue sections using the P18C5 mAb (Fig. 2b, right panel). In control sections of the rat brain (Fig. 2c), the P18C5 mAb produced intense immunolabeling in several areas (Fig. 2c, upper panel). Pre-absorption with the synthetic valine amide resulted in complete abolishment of P18C5 mAb immunostaining in adjacent brain sections (Fig. 2c, lower panel).

### P18C5 mAb immunoreactivity in the rat CNS

**Fig. 7:**
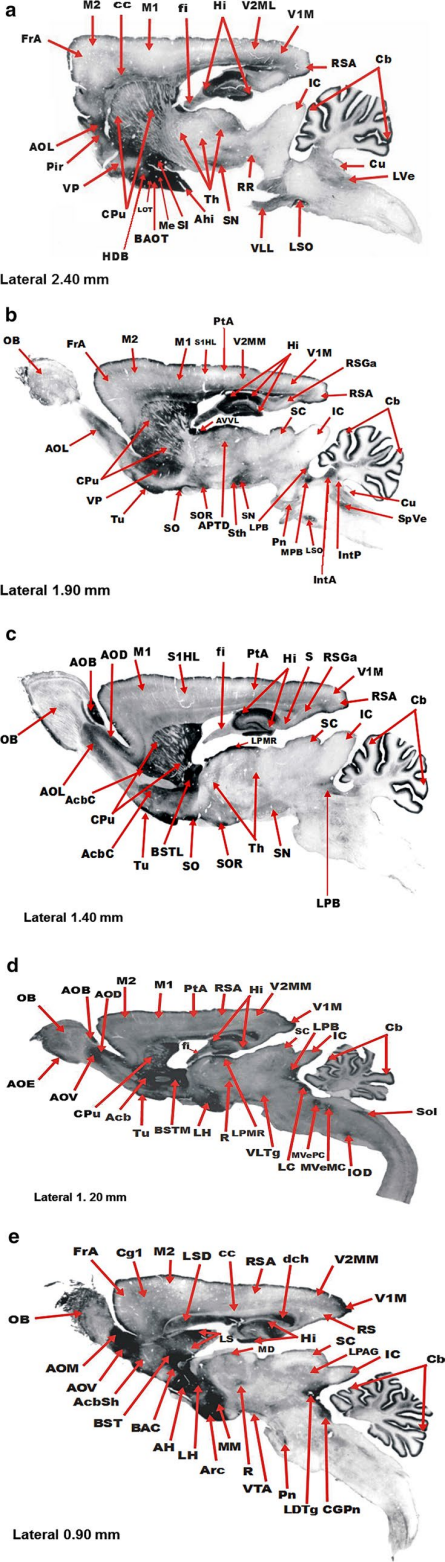
Distribution of PC18C5 mAb-ir in the rat brain. The figure illustrates the rostrocaudal distribution of PC18C5 mAb-ir in 40-μm-thick sagittal slices in fixed rat brain tissue (**a**–**e**). Bright-field photomicrographs (×40) of sagittal sections of the rat brain display lateral-to-medial aspects of the rat brain regions displaying Val-CONH_2_-ir. The neuroanatomical areas showing PC18C5 mAb-ir were identified according to the rat brain atlas of Paxinos and Watson [46]. Cortex: *FrA* frontal association cortex, *M1* primary motor cortex, *M2* secondary motor cortex, *S1HL* primary somatosensory cortex, *PtA* parietal association cortex, *V1M* primary visual cortex (monocular), *V2MM* secondary visual cortex mediomedial, *V2ML* secondary visual cortex mediolateral, *RSA* retrosplenial agranular cortex, *RSGa* retrosplenial granular A cortex, *Cg1* cingular cortex area 1–2. Olfactory system: *OB* Olfactory bulb, *AOB* accessory olfactory bulb, *AOD* anterior accessory olfactory nucleus, dorsal, *AOV* anterior accessory olfactory nucleus, ventral, *AOL* anterior accessory olfactory nucleus, lateral, *AOM* anterior accessory olfactory nucleus, medial, *AOE* anterior accessory olfactory nucleus, external, *Tu* olfactory tubercle, *BAOT* bed nucleus of the accessory olfactory tract, *LOT* nucleus of the lateral olfactory tract. Hippocampus: *Hi* hippocampal C1–C4 fields and dentate gyrus, *fi* fimbria, *S* subiculum. Basal Ganglia: *CPu* caudoputamen, *AcbC* accumbens nucleus core, *AcbSh* accumbens nucleus shell, *VP* ventral pallidum. Septum and septal areas: *BST* bed nucleus of the stria terminalis, *BSTL* bed nucleus of the stria terminalis, lateral, *BSTM* bed nucleus of the stria terminalis, medial, *BAC* bed nucleus of the anterior commissure, *LS* lateral septal nucleus, *LSD* lateral septal nucleus, dorsal part, *SI* substantia innominata, *HDB* nucleus of the horizontal limb of the diagonal band. Amygdala: *Me* medial amygdaloid nucleus, *PLCo* posterolateral cortical amygdaloid nucleus, *PMCo* posteromedial cortical amygdaloid nucleus, *ACo* anterior cortical amygdaloid nucleus, *Ahi* amygdalo-hippocampal area. Diencephalon: *Th* thalamus, *AVVL* anteroventral thalamic nucleus, ventrolateral, *STh* subthalamic nucleus (STh), *LPMR* lateral posterior thalamic nucleus, mediorostral, *MD* mediodorsal thalamic nucleus. Hypothalamus: *AH* anterior hypothalamic area, *LH* lateral hypothalamic area, *SO* supraoptic nucleus, *SOR* supraoptic nucleus, retrochiasmatic, *Arc* arcuate hypothalamic nucleus. Mammillary bodies: *MM* medial mammillary nucleus. Mesencephalon: *SN* substantia nigra, *AVT* ventrotegmental area, *VLTg* ventrolateral tegmental area, *R* red nucleus, *SC* superior colliculus, *IC* inferior colliculus, *LPAG* lateral periaqueductal gray. Pons and medulla: *LC* locus coeruleus, *LPB* lateral parabrachial nucleus, *MPB* medial parabrachial nucleus, *RR* retrorubral nucleus, *Pn* pontine nucleus (Pn), *CGPn* central gray of the pons, *LDTg* laterodorsal tegmental nucleus, *LSO* superior olive, lateral nucleus, *IOD* inferior olive, dorsal nucleus, *IntA* interposed cerebellar nucleus, anterior, *IntP* Interposed cerebellar nucleus, posterior, *Cu* cuneate nucleus, *LVe* lateral vestibular nucleus, *SpVe* spinal vestibular nucleus, *MVePC* medial vestibular nucleus, parvocellular, *MVeMC* medial vestibular nucleus, magnocellular, *VLL* ventral nucleus of the lateral lemniscus, *Sol* solitary tract. *Cb* Cerebellum. See text for additional details. *Scale bar* = ×40

Val-CONH_2_-ir was distributed widely throughout the neuroaxis of the rat brain after immunostaining using the P18C5 mAb (see the Materials and methods section) (Fig. 7a–e). Moderate to intense immunolabeling was predominantly localized to the neuropil, displaying scattered stained neuron-like cell bodies appearing in several regions within the rat brain. A brief summary of the distribution of P18C5 mAb immunoreactivity in the rostrocaudal areas of the rat CNS is described below.

### Telencephalon

Several telencephalic regions exhibited moderate to high immunolabeling, which was predominantly distributed in thin and dense fiber processes, oval-to-round somata, and diffuse fine puncta (Fig. 7a–e). PC18C5 mAb immunoreactivity (P18C5 mAb-ir) was detected in the primary and secondary motor cortices (M1 and M2, respectively), parietal sensorimotor cortical fields, and the primary and secondary visual cortices (V1M and V2MM, respectively) (Fig. 7a–e), as well as the retrosplenial agranular (RSA) and granular A (RSGa) cortices (Fig. 7b). Within the olfactory system, P18C5 mAb-ir was observed in both the main olfactory bulb (OB) and the accessory olfactory bulbs (Fig. 7c–e). In the hippocampus (Hi), P18C5 mAb-ir was found in the striatum pyramidale and radiatum, along the CA1–CA3 fields of Ammon´s horn, and, particularly, in the granular cell layer of the dentate gyrus (DG)-, as well as in the fimbria (fi) and the subiculum (S) (Fig. 7a–e). P18C5 mAb-ir was also detected in the basal ganglia [i.e., the caudoputamen (CPu), the core (AcbC) and shell (AcbSh) areas of the nucleus accumbens (NACC), and the ventral pallidum (VP)], septal areas (i.e., the stria terminalis (BST), the lateral (BSTL) and medial BST (BSTM), the bed nucleus of the anterior commissure (BAC), the lateral septum (LS), the dorsal part of the LS (LSD), the substantia innominata (SI), and the nucleus of the horizontal limb of the diagonal band (HDB)] and the amygdala [anterior (Aco), including the posterolateral (PLCo), and posteromedial cortical amygdaloid nuclei (PMCo)], which displayed moderate to high immunostaining that was predominantly distributed in sparse neuropil, stained round-to-oval cell bodies, and fine dendritic puncta (Fig. 7a–e). Both the medial amygdaloid nucleus (Me) and the amygdalo-hippocampal area (Ahi) exhibited low levels of mAb-ir (Fig. 7a).

### Diencephalon

P18C5 mAb-ir was observed in several diencephalic structures, including the hypothalamus [i.e., the anterior hypothalamic (AH) and lateral hypothalamic areas (LH) and the supraoptic (SO) and arcuate nuclei (Arc)], which displayed moderate to intense mAb immunostaining, (Fig. 7d–e). Both the thalamus (Th) i.e., the anteroventral (AVVL), lateral posterior (LPMR) and mediodorsal nuclei (MD)] and the subthalamic nuclei (STh) (Fig. 7a–c), including several mammillary nuclei i.e., the medial mammillary (MM), lateral mammillary (ML), and submammilothalamic nuclei (SThM)], displayed low to moderate mAb immunolabeling (Fig. 7a–e). The stained regions included the retrochiasmatic supraoptic (SOR) nucleus of the hypothalamus (Fig. 7a–c).

### Mesencephalon

Along the mesencephalon, P18C5 mAb-ir was detected in the substantia nigra (SN, pars compacta, pars reticularis, and pars lateralis) (Fig. 7a–c), the superior colliculus (SC) [i.e., the zonal layer (Zo)], the inferior colliculus (IC) (i.e., the central nucleus) (Fig. 7c–e), the ventral tegmental area (VTA), the red nucleus (R) and the lateral periaqueductal gray area (LPAG) (Fig. 7e). These mesencephalic areas generally displayed low to moderate mAb immunolabeling that was predominantly localized within a network of fiber processes dispersed among stained somata (Fig. 7a–e).

### Cerebelum

PC18C5 mAb-ir was predominantly distributed along the Purkinje cell layer of the cerebellar cortical layers; cells in this area displayed highly stained somata-like cell bodies, fine dendritic puncta, and a very dense network of protruding labeled Purkinje cell axon fibers (Fig. 7a–e). Low to moderate mAb-ir was detected in both the flocculus (fl) and the white matter of the Cb, including the medial cerebellar peduncle (mcp) (data not shown).

### Pons and medulla

The PC18C5 mAb showed low to moderate immunostaining in nuclei and tracts along the rostrocaudal aspects of the pontine and medullary areas of the rat CNS (Fig. 7a–e). Within the most lateral aspects of the pontine/medullary areas of the brain, low to moderate mAb-ir was detected at the retrorubral nucleus (RR), the ventral nucleus of the lateral lemniscus (VLL), the lateral nucleus of the superior olive (LSO), the cuneate nucleus (Cu), and the lateral vestibular nucleus (Lve) (Fig. 7a). In addition, the pontine nucleus (Pn), both the medial (MPB) and lateral parabrachial nuclei (LPB), both the anterior (IntA) and posterior parts of the interposed cerebellar nuclei (IntP), and the spinal vestibular nucleus (SpVe) showed positive immunostaining (Fig. 7b–c). Within the medial aspects of these caudal regions of the rat brain, positive mAb-ir was detected at the locus coeruleus (LC), the parvocellular (MvePC) and magnocellular parts of the medial vestibular nuclei (MveMC), and both the laterodorsal tegmental nucleus (LDTg) and the dorsal part of the inferior olive (IOD), including the central gray matter of the pons (CGPn) and the solitary tract (Sol) (Fig. 7d–e).

### Valine amide peptide immunoreactivity in the rat brain

The reports describing the immunoreactive distribution of α-MSH, metorphamide, secretin and UCN1-2 in the rat brain [32–36] were used to compare each peptide immunoreactivity with the rostrocaudal distribution of P18C5 mAb-ir in the rat CNS, as depicted in Fig. 7a–e, respectively.

### Subtraction analysis

P18C5-mAb-ir detected along the rostrocaudal areas of the rat brain (Fig. 7a–e) was compared with the distribution of valine amide peptide immunoreactivities detected in the rat brain. The immunoreactive signals obtained for each peptide were subtracted from PC18C5 mAb-ir signals detected along the neuroaxis of the rat brain. The subtracted brain areas expressing Val-CONH_2_-ir material are depicted in Fig. 3. Most of these areas showed moderate-to-high PC18C5 mAb-ir signals, with the exception of the cerebellar lobes (i.e., the anterior, posterior and flocculonodular lobes) and the lateral hypothalamus (LH) which exhibited an intense P18C5-mAb-ir signals, similar to immunoreactive signal displayed by the IL of the hypophysis, used as the internal control tissue.

As shown in Fig. 3, valine amide peptide immunoreactivity (VAP-ir) was detected in cortical areas of the *telencephalon* such as M2, the hindlimb (S1HL) and trunk regions of the primary somatosensory cortex (S1Tr), the parietal association cortex (PtA), V2MM, and V2ML. In addition, CVAP-ir was observed in the main accessory OB (AOB) and corresponding nuclei, described as the anterior accessory olfactory nucleus dorsal (AOD), the nucleus ventral (AOV), the nucleus lateral (AOL) and the nucleus medialis (AOM). Furthermore, VAP-ir was detected along the olfactory tubercle (Tu), within the bed nucleus of the accessory olfactory tract (BAOT) and within the nucleus of the lateral olfactory tract (LOT). In addition, CVAP-ir was detected in telencephalic structures such as S of the Hi, NACC, and the substantia innominata of the LS, including both the Ahi and the amygdalo-anterior area (AA) of the limbic system. In the *diencephalon,* VAP-ir was found in LPMR of the thalamus and in the magnocellular portion of the LH. Within the *mesencephalon*, VAP-ir was primarily localized to the lateral portion of the substantia nigra (SNL) and Zo of the SC, whereas in the *pontine* and *medullary* areas, CVAP-ir was primarily localized to RR, Sol and the fl of the Cb.

### Peptide fractions displaying VAP-ir

**Fig. 8:**
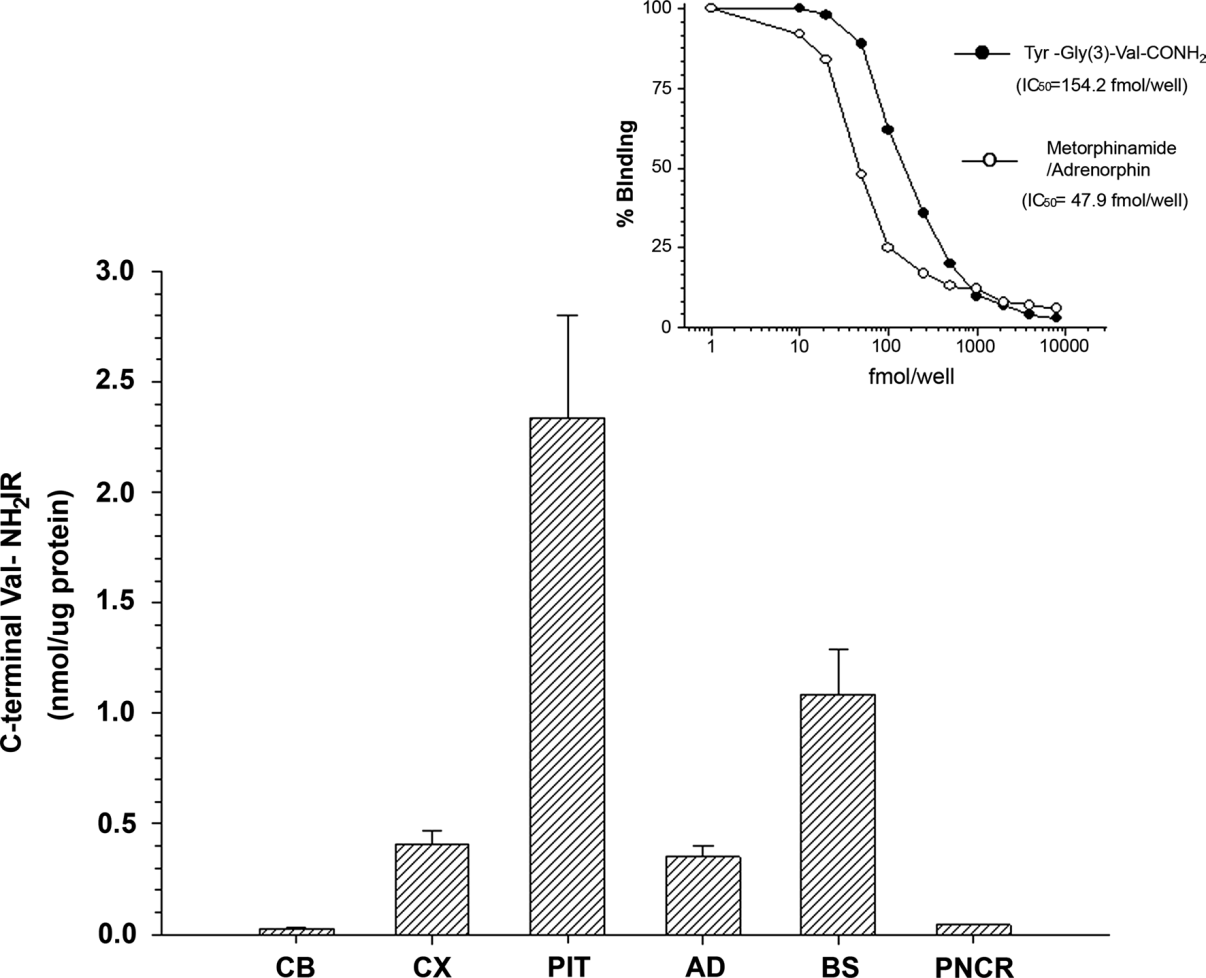
Quantification of endogenous valine amide peptide-immunoreactive material in tissues. The abundance of valine amide peptide-immunoreactive material was estimated in purified peptide fractions prepared from subtracted brain regions (CB, CX, BS) and neuroendocrine glands (PIT, PNCR), which displayed moderate-to-high PC18C5 mAb immunolabeling (see Figs. 2 and 7) based on the solid-phase RIA developed for the PC18C5 mAb (inset). The plot shows that the highest abundance of Val-CONH2-ir material was found in the pituitary (PIT) (2.34 ± 0.4672 nmol/μg protein; mean ± SEM), followed by the brainstem (BS) (1.09 ± 0. 021 nmol/μg protein; mean ± SEM), the adrenal gland (AD) (0.35 ± 0.052 nmol/μg protein; mean ± SEM), and the cortex (Cx) (0.41 ± 0.06 nmol/μg protein; mean ± SEM). Inset depicts a representative solid-phase RIA for the PC18C5 mAb used for the quantification of valine amide-immunoreactive material in the assays. Tyr-Gly(3)-Val-CONH_2_ was used as a standard to construct a typical standard curve and was detected at a concentration of 154.2 fmol/well at the IC_50_ value, whereas metorphamide/adrenorphin (used as a competitive peptide antigen) was detected at a concentration as low as 7.9 fmol/well at the IC_50_ value (see text for additional details)

Several regions of the rat brain and endocrine glands were used to prepare purified peptide fractions from the subtracted brain areas to quantify the content of VAP-ir (expressed as nmol/μg protein) using the specific solid-phase RIA generated for the P18C5 mAb (Fig. 6). In addition, neuroendocrine glands (pituitary, adrenal and pancreas) were used as control tissues in the assay. As shown in Fig. 8, the rat pituitary (PIT) displayed the highest abundance of VAP-ir (2.34 ± 0.4672 nmol/μg protein; mean ± SEM), which was 2.1-fold greater than the amount observed in the brainstem (1.09 ± 0.21 nmol/μg protein; mean ± SEM). However, low amounts of Val-CONH_2_-ir were detected in the cortex (0.41 ± 0.06 nmol/μg protein; mean ± SEM) and in the adrenal glands (AD) (0.35 ± 0.052 nmol/μg protein; mean ± SEM); these levels were 6.7- and 14.6-fold lower, respectively, than the levels measured in the pituitary. In the same context, both the Cb (0.026 ± 0.004 nmol/μg protein; mean ± SEM) and the pancreas (0.042 ± 0.005 nmol/μg protein; mean ± SEM) contained low levels of Val-CONH_2_-ir, specifically 90- and 56-fold lower, respectively, than those in the pituitary.

## Discussion

The α-amidation process is an essential step for the conversion of prohormones into mature peptides that possess complete biological activity, which includes receptor recognition and transduction of peptide hormone signals in target cells [4, 5, 10]. The α-amidation process has been shown to occur most frequently on neutral amino acids (L, F, G) in neuropeptides, which constitute the largest group (>50 %) of functional peptide messengers identified in vertebrate and/or invertebrate species. Such peptides display pleiotropic activities across species [2, 3, 16]. However, valine amide peptide hormones represent the smallest group of functional amidated peptides identified to date, and which include the enterochromaffin cell-related peptide hormone, *secretin*, which belongs to the PACAP/VIP/glucagon peptide hormone family [22, 50]; the POMC-derived peptide- *α*-*MSH* - [47]; the proenkephalin A-derived *metorphamide/adrenorphin* [48]; the stress and appetite regulatory *urocortin*s (*UCN1*-*2)* [23, 49] and the *enterins,* a family of brain peptides found in the enteric nervous system of Aplysia, which modulate both feeding non-feeding behaviors in this marine specie [24].

In order to search for neural structures expressing putative novel valine amide peptide immunoreactive material in the brain and neurosecretory tissues of mammals, we employed splenocyte/myeloma cell fusion procedures, with the aim to generate stable hybridoma-producing mAbs able of recognizing the structural α-amidated isoform of the valine residue (Val-CONH_2_) in constructed immunoconjugates (see material and methods and Figs. 5 and 6).

Cell fusion procedures between activated splenocytes from the hyperimmune female BALB/c mouse -R4—(Fig. 4) and the murine myeloma cell line Sp2/0, led to the identification of three hybridoma-producing mAbs (out of 340 screened hybridoma-forming colonies) designated as P15A4, P17C11, and P18C5 and which displayed different levels of cross-reactivities against the structural α-amidated and/or the free carboxylic acid isovariants of the valine residue expressed in distinct antigens tested in different immunoassays (ELISA, dot–blots and solid-phase RIA) used to validate the specificity and selectivity of these mAbs (see Figs. 5 and 6). The efficiency of the cell fusion procedure from the total cells seeded for fusion was 0.016 %, a value considered within the efficient rates reported in the generation of productive hybridomas [41, 42].

As shown, only P18C5 mAb showed a predominant cross-reactivity against the α-amidated isoform of the valine residue in the tested antigens in ELISA and dot-blot assays (Fig. 5) However, P18C5 mAb showed to be exquisitely selective for the structural α-amidated isoform of the valine residue in the constructed synthetic antigens (Methorphamide, BSA-Val-CONH_2_, Tyr-Gly_2-4_-Val-CONH_2_) tested in the fmol-sensitive solid phase RIA (Fig. 6) but unable to recognize the free carboxylic acid of same residue in both RDGV, Tyr-Gly_2-4_-Val-COO^−^ peptide antigens (within the range of IC_50_ values detected for the valine amidated antigens, including Mastoparan X [a tetradecapeptidetoxin from wasp venom containing the structural α-amidated isoform of the Leu residue (−Leu–NH_2_)] [51].

The high specificity and selectivity exhibited by P18C5 mAb in recognizing the condensed amide (−NH_2_) substituent at the carbonyl group (−CO–NH_2_) of the asymmetric carbon (Cα) in the amino acid, may be due to the sort of complementarity determining regions (CDRs) encoded along both the heavy and light chains of the antibody, enhancing its preferential affinity and recognition for the structural amide (−NH_2_) epitope over the functional carbonyl group (–C = O) of the amino acid [29, 30]. In same line, specific mAbs were shown to display predominant specificities and selectivity in the recognition of small antigens (≤0.3 kD) such as, acetyllysine and –methyllysine antigenic epitopes in proteins [31], or mAbs shown to discriminate among isovariants (α, β, γ, and δ) of the immune mediator, interleukin, IL-32 [30].

Based on our immunoassay results, we explored the capability of the P18C5 mAb to detect valine amide peptide immunoreactivity along the neuroaxis of the rat brain and rat neuroendocrine tissues (pituitary and adrenals) (Figs. 2 and 7). Our results showed that P18C5 mAb to was able to recognize the structural α-amidated isoform of the valine residue in endogenous peptide material, as demonstrated for abolishment of mAb immunostaining in tested tissues (Fig. 2a–c) after preadsorbing the antibody with the synthetic valine amidated residue (Val-CONH_2_) (see “Materials and methods”). As shown in Fig. 2, the intermediate lobe (IL) of the pituitary (used as the internal control for the detection of immunoreactive signals in along the neuroaxis of the rat brain (Fig. 3) showed an intense mAb immunostaining when compared to absence of immunolabeling in both AL and the PL in same neuroendocrine tissue (Fig. 2a). This neurosecretory area contains the “pituitary melanotrophs” shown to synthesize and release high concentrations of POMC-derived peptides (β-endorphin, ACTH, and α-MSH) into the bloodstream [47]. Thus, the detection of P18C5 mAb-ir in the IL of the hypophysis reflects the post-translational processing of POMC-derived peptides and conversion of mature C-terminal amidated peptide products, via the activity peptidylglycine α-amidating monooxygenase (PAM) [4–6, 47]. In same line, the detection of P18C5 mAb-ir at the core (*me*) of the adrenal gland supports the cellular expression of bioactive metorphamide/adrenorphin peptide released from the proenkephalin A protein precursor from the chromaffin cells [48, 52].

P18C5 mAb-immunoreactivity showed a wide distribution along the neuroaxis of the rat brain (Fig. 7a–e). Most of the rostrocaudal areas expressing a low-to moderate or conspicuous immunostaining were matched with brain areas previously shown to contain the most prevalent bioactive valine amide neuropeptides α-MSH (α-MSH) [33]; secretin (Sec) [35]; adrenorphin (Met) [34] and UCNs (UCN1-2) [36], suggesting that these peptides exert pleiotropic effects in time and tissue-specific manner, in both central and peripheral tissues after targeting their specific cell surface receptors [54, 55]. The properties and functions of valine amide peptide in the brain and peripheral tissues may be search in the web and in some reviews described herein [2, 47, 50, 53–56] and is out of the scope of the present paper to detail the relationship between brain structure and valine amide peptide bioactivities.

Data obtained from the subtraction analysis revealed the expression of mAb-immunolabeling in specific brain areas that did not match with the neuronal distribution of valine amide neuropeptides (Fig. 3). These results suggest that such areas represent neural structures able to synthesize putative novel valine amidated peptide products encoded in large propeptide precursor protein(s) with functions yet to be elucidated. Furthermore, these areas suggest that neurons operating within each immunolabeled brain region or nuclei must express same molecular machinery and posttranslational enzyme-dependent mechanisms, in order to allow the biosynthesis and conversion of prohormones into functional C-terminal amide neuropeptides [1–3, 19, 47, 49, 50] at the expense of the functional bioactivities of PC1/PC3 proconvertases [47, 57] and the peptidylglycine α-amidating monooxygenase (PAM) [4], respectively.

This hypothesis is supported by the specific tissue-dependent processing of POMC-derived peptide hormones (α-MSH, ACTH, β-endorphin) [47] in the brain and peripheral tissues [57], including the processing of proenkephalin A in the brain and neurosecretory tissues such as, the chromaffin cells of the adrenal medulla (me) [52].

Moreover, based on the specificity and selectivity of the P18C5 mAb for detecting immunoreactive peptide material in the rat brain and neuroendocrine tissues; we explored the feasibility of this mAb to determine the abundance of Val-CONH_2_-ir in peptide fractions isolated from immunostained subtracted regions using our fmol-sensitive solid-phase RIA raised for P18C5 mAb (Fig. 6). As shown, both cortex (Cx), brainstem (BS) and Cb were used as representative subtracted brain areas encoding putative valine amide peptide material, in addition of the pancreas, the pituitary and adrenal glands, used as neuroendocrine control tissues (Fig. 8).

Furthermore, the highest amount of Val-CONH_2_-ir peptide material was detected in the pituitary (PIT) followed by the brainstem (BS), the adrenal gland (AD) and the cortex (Cx) (Fig. 8). The relative abundance of immunoreactive peptide material determined in both BS, AD and the Cx were 46.6, 15 and 17.5 %, respectively, from that detected in the pituitary. In same line, both cerebellum (Cb) and pancreas (PNCR) showed lowest content of estimated Val-CONH_2_-ir (1.1–1.8 %). Differences in the relative amount of peptide immunoreactivity estimated for each neural tissue could rely on the cell concentration of valine amide neuropeptides stored (in secretory granules) before their release into the extacellular milieu [6, 58] at the time of tissue extraction and preparation of peptide fractions from tissue homogenates. Although at this stage during mAb screening, the exact amount of putative novel valine amide neuropeptides per tissue (in the case of their existence) may be impossible to obtain. However, both recombinant DNA technology and cloning procedures [27] may lead to the identification and characterization of putative novel cDNAs encoding valine amide peptide sequences, as demonstrated for the “*enterins*”, a family of structurally related nonapeptides and decapeptides that are present in the gut and CNS of Aplysia, and most of which share the HSFVamide sequence at the C terminus [24].

## Conclusions

The present work describes an experimental strategy used to generate murine monoclonal antibodies able to differentiate between two isovariants of the valine residue. One single mAb designated as, P18C5 mAb, showed a unique specificity and selectivity for the structural α-amidated isoform of this neutral amino acid in natural (metorphamide/adrenorphin) and synthetic (–Val-CONH_2_) peptide antigens, as revealed from our immunoassays, besides of demonstrating a great capability to detect putative—yet unidentified—valine amide peptide immunoreactive material in specific areas of the rat brain. The ability of this mAb to distinguish between two small structural epitopes in same residue, posit for its wide applications in both research and clinical settings. For instance, P18C5 mAb may be used to generate specific biological “kits” used to purify and detect valine amidated peptide products in tissue and/or biological fluids. Furthermore, this mAb may be used in immunotherapy settings to reduce tumor growth and tumorigenesis; or used as a detoxifying–neutralizing reagent from blood poisoning compounds such as, toxic amidated peptide molecules found in food, plants and/or animal species. Overall, this work portraits the potential applications of a single mAb, who displays exquisite properties when reacting against valine amide peptide antigens, including its capability to identify brain regions expressing putative novel valine amide neuropeptides, with functions yet to be discovered.
